# Scale of Emotional Development‐Questionnaire: A Systematic Approach to Improving Performance

**DOI:** 10.1111/jir.70019

**Published:** 2025-08-20

**Authors:** Mitchel Mesker, Jolanda Vonk, Suzanne D. M. Derks, Veerle Andries, Angelique van Lier‐Weir, Paula S. Sterkenburg

**Affiliations:** ^1^ Department of Clinical Child and Family Studies Vrije Universiteit Amsterdam Amsterdam the Netherlands; ^2^ ORO/Lore Behandelcentrum Helmond the Netherlands; ^3^ Stichting Odion Wormer the Netherlands; ^4^ Bartiméus Doorn the Netherlands

**Keywords:** emotional development, intellectual disability, interview, mental health, questionnaire

## Abstract

**Background:**

In people with intellectual disabilities, emotional development progresses more slowly or stagnates, which can result in challenging behaviours. The Scale of Emotional Development‐Questionnaire was designed to chart people's own emotional development, providing insight into basic emotional needs and resilience, while reducing prejudice, enhancing self‐awareness and improving emotional expression.

**Methods:**

The questionnaire was completed by 134 participants with moderate to borderline intellectual disabilities. Reliability, validity, internal structure and item performance were analysed to identify areas for improvement.

**Results:**

Preliminary analyses indicated the questionnaire captures key aspects of emotional development, with most items showing strong factor loadings (51.79%). However, multiple items may require refinement due to moderate loadings (30.00%), low loadings (18.21%), limited variance (3.93%) and negative‐low correlations.

**Conclusion:**

The Scale of Emotional Development‐Questionnaire is a promising self‐report interview of emotional development, complementing the proxy perspective of the Scale of Emotional Development‐Short. The findings highlight areas for improvement and the need for further research post‐revision.

## 1 Introduction

Human development typically progresses through developmental stages, both physically and mentally, culminating in adulthood when the brain matures. However, for people with atypical development, such as moderate to borderline intellectual disabilities characterised by an intelligence quotient ranging from 35 to 85, this progression may follow a different trajectory (American Psychiatric Association 2013; de Bruijn et al. 2017; Schalock and Luckasson 2014). In such cases, emotional development may progress more slowly and often stagnates at an earlier stage (Sappok et al. 2022). This raises an important question: At what point does emotional development stagnate? Emotional development (socio‐emotional brain functions) is defined as ‘the ability to regulate one's own emotional expression, to identify the emotional expressions of others, to interpret emotional cues, to respond accordingly, and to self‐soothe and manage emotional outbursts’ (Sappok et al. 2022, p. 3).

For people with intellectual disabilities, emotional development often lags behind their cognitive abilities, resulting in emotional needs that do not align with their cognitive abilities. This misalignment can create a mismatch between environmental demands and a person's capacity to cope, leading to stress. Increased stress can further exacerbate this discrepancy, causing regression to earlier emotional stages and altering basic emotional needs. As these needs shift, the gap between environmental demands and people's capacity to cope widens, amplifying stress even further (Sappok et al. 2013; Sappok et al. 2014).

By tailoring support to the interplay of emotional and cognitive functioning, caregivers can better meet people's needs without exceeding their capacities (Dosen 2005a, 2005b). This involves adapting social interactions and environments to align with a person's emotional developmental level, ensuring that expectations, communication and daily activities are appropriately structured. Barrett et al. (2024) found this approach reduced psychotropic drug and antipsychotic use, highlighting its effectiveness. In contrast, when support fails to align with a person's emotional needs, it can increase stress for both the caregiver and the client (Lou et al. 2019), leading to behavioural challenges, frustration and a decline in quality of care (Lou et al. 2019; Sappok et al. 2014). Prolonged stress may result in burnout and even psychiatric conditions, such as oppositional‐defiant disorder (Dosen 2005a, 2005b). Therefore, understanding emotional development is crucial for interpreting behaviour, promoting higher‐quality care and fostering more stable caregiver–client relationships.

To ensure that care and support are effectively tailored to a person's emotional development, a reliable and adequate assessment tool is essential. Several instruments have been developed to measure and describe emotional development, including the Scale of Emotional Development‐Short (SED‐S; Sappok et al. 2023). The SED‐S is a widely used and reliable instrument in healthcare settings that utilises a semi‐structured proxy interview to assess emotional development (Sappok et al. 2016).

While proxy‐based instruments like the SED‐S are valuable, growing attention has been given to self‐report approaches that align more closely with principles of inclusion and respondent empowerment (Kooijmans, Mercera, et al. 2022). Self‐report instruments are critical for capturing authentic individual experiences, particularly in domains that are subjective or difficult to observe, such as emotional well‐being, cognition and social interactions (Li et al. 2015). Self‐reporting reduces bias introduced by proxy respondents, whose interpretations may differ from the individual's own experiences, as proxies, especially non‐spouses, rate subjective experiences less positively (Elliott et al. 2008). Even limited involvement, such as reading or translating questions, can affect the authenticity of responses (Elliott et al. 2008; Kooijmans, Langdon, and Moonen 2022). Retrospective proxy reports are only moderately associated with self‐reports and are shaped by the proxy's own emotional stage, highlighting the need for accurate, person‐centred self‐reporting (Infurna et al. 2014). Together, these findings underscore the necessity of self‐report methods.

To address the need for more person‐centred assessments, the Scale of Emotional Development‐Questionnaire (SED‐Q; Vonk 2021) was developed as a semi‐structured self‐report interview in which the client responds and the professional evaluates the answers, using probing questions to elicit further information, to determine the presence of specific aspects of emotional development, with a focus on self‐reported emotional experiences. This approach acknowledges that many people with intellectual disabilities are capable of expressing their own emotional experiences (Kooijmans, Mercera, et al. 2022). The SED‐Q extends the reference age to 25 years by incorporating two additional developmental stages, enhancing its applicability and utility (Vonk 2021). To ensure comparability, the SED‐Q was adapted by two psychologists for people with intellectual disabilities and aligned with the SED‐S (2016), previously validated by Flachsmeyer et al. (2023).

In addition to emotional development, mentalisation and epistemic trust provide complementary perspectives. They are distinct psychological constructs, yet they remain closely interconnected (Fonagy and Allison 2014; Knapen 2024). Mentalisation refers to the skill of understanding oneself and others through the lens of mental states, including emotions, beliefs and desires (Bateman and Fonagy 2006). Epistemic trust, on the other hand, is the willingness to regard new information from others as generalizable, reliable and relevant to the self (Fonagy and Allison 2014). Given the interconnectedness of these constructs (Knapen et al. 2025), it becomes essential to investigate whether instruments designed to measure them can effectively distinguish between them.

This preliminary instrument evaluation, or pilot study, aims to explore the initial psychometric properties of the SED‐Q, specifically its internal structure, reliability and preliminary validity in assessing emotional development. Unlike a full‐scale validation study, this study primarily seeks to identify areas for improvement. As part of evaluating preliminary construct validity, this study also investigates known‐group validity by comparing outcomes across groups that theoretically differ in emotional development. Specifically, differences are anticipated between people with varying severities of intellectual disabilities, as well as co‐occurring autism (Sappok et al. 2020), while no differences are expected for people with visual impairments (Sterkenburg et al. 2022). Furthermore, given that the SED‐Q relies on interviewer administration, this study also examines whether interviewer experience might influence the results. The insights gained from these preliminary findings will guide necessary refinements, laying the groundwork to conduct a comprehensive validation study.

## 2 Methods

### 2.1 Participants

Participants (*N* = 148) aged 18 years or older, with a moderate to borderline intellectual disability with or without a visual impairment, were eligible to participate. After examining the SED‐Q baseline data, 134 participants were included, while 14 were excluded for incomplete questionnaires. Table 1 shows the demographic characteristics of the included participants.

**TABLE 1:** Baseline demographic characteristics (*n* = 134).

Variables	Total
Gender	
Male (%)	56 (41.79%)
Female (%)	77 (57.46%)
Intelligence quotient	
Borderline intellectual disability (71–85 IQ)	18 (13.43%)
Mild intellectual disability (50–70 IQ)	94 (70.15%)
Moderate intellectual disability (35–49 IQ)	14 (10.45%)
I do not know^a^	5 (3.73%)
Impaired vision	32 (23.88%)
Visually impaired	12 (8.86%)
Severely visually impaired	9 (6.72%)
Blind	11 (8.21%)
Age in years *M* (SD)	40.90 (14.45)
Autism diagnosis (%)	25 (18.66%)
Autistic traits *M* (SD)	3.59 (1.48)

*Note:* Age, autistic traits and personal well‐being are reported as means (*M*) and standard deviations (SD). Percentages are calculated based on available data for each variable. Percentages may not add up to 100% due to missing data.

^a^ Recruitment was conducted via care facilities for people with (visual and) intellectual disabilities. Caregivers were asked to report the participant's intellectual disability, but some were unable to do so. As the participants received supporting care, they were included in the study regardless.

### 2.2 Power Analysis

Table 2 displays an overview of the statistical power associated with each main analysis.

**TABLE 2:** Statistical power analyses.

Analysis type	Test details	Parameters	Effect size	Power	Interpretation
Pearson's correlation	G*Power 3.1.9.6, two‐tailed (Faul et al. 2007)	*n* = 134, *α* = 0.05	*r* = 0.30 (medium)	94%	Adequate power
ICC for test–retest reliability	Web‐based ICC calculator (Arifin 2018)	Expected ICC = 0.70; α = 0.05 Recommended: 79–101; actual: 43		Underpowered	Insufficient sample size for adequate or precise ICC
ANOVA—intellectual disability	R (pwr package; Champely 2020), 4 groups	*n* = 134, α = 0.05	*f* = 0.36 (medium–large)	0.95	Adequate power
ANOVA—autism diagnosis	R (pwr package; Champely 2020), 2 groups	*n* = 134, α = 0.05	*f* = 0.01 (negligible)	0.05	Underpowered
ANOVA—interview experience	R (pwr package; Champely 2020), 2 groups	*n* = 134, α = 0.05	*f* = 0.11 (small)	0.16	Underpowered
ANOVA—visual impairment	R (pwr package; Champely 2020), 3 groups	*n* = 134, α = 0.05	*f* = 0.11 (small)	0.20	Underpowered
ANOVA—ID by domain	R (pwr package; Champely 2020), 3 groups	*n* = 134, α = 0.05	Domains 7 and 8: *f* ≈ 0.35	0.89–0.91	Sufficient power for Domains 7 and 8; others ranged 0.23–0.59

*Note:* Effect size interpretations follow Cohen's (1988) guidelines. Data sources: Arifin (2018), Champely (2020) and Faul et al. (2007).

Abbreviations: *f* = effect size for ANOVA; ICC = intraclass correlation coefficient; ID = intellectual disability; *n* = sample size; α = alpha level.

## 3 Materials

### 3.1 Participant Attributes

#### 3.1.1 Minimal Dataset (MDS)

MDS is used to gather demographic information. ‘Basic MDS fixed, for adults’ and ‘basic MDS for adults with an intellectual disability’ are self‐report questionnaires used to gather biographical data such as age and gender (Kunseler et al. 2019). An example item is: “In which country are you born?” (Kunseler et al. 2019). Additional to the MDS, participants are asked about the presence and severity of visual impairments. Furthermore, professional caregivers are asked about the severity of the intellectual disability of the participant.

#### 3.1.2 Autism Spectrum Quotient (AQ‐10)

The AQ‐10 is a self‐assessment tool for identifying autistic traits. An adapted version of the AQ‐10 was used for adults with mild to borderline intellectual disabilities. This adapted version included ‘Yes’, ‘No’ and ‘I don't know’ as response format, along with adjusted questions tailored to the target group. The AQ‐10 consists of 10 items. Respondents scored 1 point for answering ‘Yes’ on items 1–5 and 10. For Items 6–9, a score of 1 point is awarded if the response is ‘No’. Item 8 was excluded due to issues with administering the questionnaire. The total score ranges from 0 to 9, calculated by summing the individual scores, with higher scores indicating more autistic traits. An example item is, ‘I often hear sounds that other people don't notice’ (Kent et al. 2018, Appendix A). The adapted version demonstrated fair reliability with a Cronbach's alpha of 0.67 (Allison et al. 2012; Kent et al. 2018), but showed unsatisfactory reliability in the current study with a Cronbach's alpha of 0.50.

### 3.2 Emotional Development

#### 3.2.1 SED‐Q and SED‐S

The SED‐Q is a semi‐structured self‐report interview conducted by a professional, designed to assess levels of emotional development (Vonk 2021). It is based on the SED‐S (Sappok et al. 2016; Tarasova et al. 2022) but uses the participant's own responses. The SED‐Q includes 280 items, structured into eight domains representing essential aspects of emotional development. Domains span areas such as (1) self‐awareness, (2) relationships with significant others, (3) adaptation to new environments, (4) emotion regulation, (5) peer interactions, (6) use of leisure and materials, (7) communication and (8) stress management. Each domain is divided into seven stages, reflecting developmental characteristics. These stages are adaption, socialisation, first individuation, identification, reality awareness, social autonomy and social responsibility (Flachsmeyer et al. 2023; Vonk 2021). This semi‐structured self‐report interview conducted by a professional allows for active facilitation by a trained professional, who guides the individual through the interview process using probing questions and developmentally appropriate language. The trained professional is responsible for interpreting and rating the individual's responses. Each stage within a domain comprises items that are evaluated in a binary format, designated as either ‘characteristic’ or ‘not characteristic’. The score for each domain being the stage with the highest number of ‘yes’ responses, in case of a similar score the lowest level of emotional development is selected. The results from the domains are ordered from low to high, with the fourth lowest determining the level of emotional development. Higher scores indicate a more advanced level of emotional development. An example item is: ‘How do you handle stimuli?’

The SED‐S is a proxy‐administered version designed for people with intellectual disabilities (Sappok et al. 2016). It contains 200 items and assesses emotional development up to 12 years of age across five stages. Items are also rated in a binary format, with scoring following the same principle as the SED‐Q. An example item is: ‘He passively enjoys sensory stimuli’ (Sappok et al. 2016, p. 170). A large international study by the Network of Europeans on Emotional Development (NEED) between 2016 and 2021 demonstrated that the SED‐S has excellent reliability with a Cronbach's alpha of 0.93 (Flachsmeyer et al. 2023). This study used the validated 2016 version, as Stage 6 was developed concurrently and unvalidated, demonstrating excellent reliability with a Cronbach's alpha of 0.91 (Tarasova et al. 2022).

### 3.3 Additional Questionnaires

#### 3.3.1 Reflective Functioning Questionnaire (RFQ)

The RFQ is a self‐report questionnaire developed to assess reflective functioning, defined as ‘the capacity to understand ourselves and others in terms of intentional mental states’ (Allen et al. 2008, p. 348). An adapted version of the RFQ was used for people with mild to borderline intellectual disabilities (Derks et al. 2023). The RFQ consists of 10 items, measured on a 7‐point Likert scale from 1 (*strongly disagree*) to 7 (*strongly agree*). The final score is calculated by reverse scoring Item 8, then averaging items. Scores range from 1 to 7, indicating increasing uncertainty about mental states. A moderate score is ideal, representing balanced certainty. An example item is: ‘I don't understand what people think’ (Derks et al. 2023, result section). The RFQ includes two subscales: the first centres on the self (subscale Self; 2–7) and the second explores the self in relation to others (subscale Other; 1, 8–10). Comparable to the study of Derks et al. (2023), the RFQ showed fair to moderate reliability with Cronbach's alphas of 0.76 (total), 0.75 (Self) and 0.61 (Other).

#### 3.3.2 Questionnaire Epistemic Trust (QET)

The QET is a self‐report questionnaire designed to assess epistemic trust (Fonagy and Allison 2014). The QET includes 24 items scored on a 5‐point Likert scale ranging from 1 (*totally agree*) to 5 (*totally disagree*). The overall score range from 24 to 120 and obtained by reverse‐scoring negatively phrased items (1–5, 10–13, 16, 18–20, 24) and summing responses, with higher scores indicating greater epistemic trust. An example item is: ‘I am interested in what my therapist can teach me’ (Knapen et al. 2024, p. 7). The QET is divided into four subscales: Hypervigilance (Items 1–6), Curiosity/Openness (Items 7–9, 21–23), Expectation of Help (Items 10, 12, 14–17) and Openness to Help (Items 11, 13, 18–20, 24). The initial study demonstrates excellent internal consistency, with a Cronbach's alpha of 0.91 and good to excellent for the subscales ranging from 0.80 to 0.90 (Knapen et al. 2024) and good reliability (0.82) in this study.

### 3.4 Procedure/Data Collection

Participants were recruited from Dutch and Flemish care organisations providing care for people with intellectual and/or visual disabilities, between December 2022 and December 2023. Additionally, some participants were recruited through social networks (e.g., client councils, online platforms). People who did not understand Dutch or were seriously ill (e.g., hospitalised) were excluded. An information brochure, co‐created with adults with intellectual disabilities, was developed to ensure accessibility. The brochure, along with an adapted version featuring visual aids (e.g., images, audio recordings or videos) and an information letter, was provided to potential participants, their supervisors, parents and other caregivers for recruitment.

Independent researchers received 3 h of training on administering the SED‐Q and SED‐S. They collected and assisted participants with questionnaires, following a detailed instruction manual. Responses were anonymised and collected via Qualtrics or, if objected to, on paper and manually entered into the database. Data was securely stored on the Research drive, complying with the Dutch Personal Data Protection Act and American Psychological Association regulations, with access restricted to project researchers.

Participants received reminders about their appointments a few days prior. Independent researchers conducted questionnaires and interviews at participants' locations, ensuring a quiet and comfortable setting. The first appointment lasted 90 min for participants (with breaks if needed) and 60 min for their supervisors, parents or caregivers. Participants were invited to take part a second time 3 weeks later, with the follow‐up taking approximately 45 min. Forty‐three participants completed the follow‐up. Participants were given a small gift upon completing their participation.

### 3.5 Data Analysis

The data analysis was conducted using R Version 4.3.0 (R Foundation for Statistical Computing, Vienna, Austria) within RStudio (R Core Team 2024). A significance level of *α* = 0.05 was applied throughout.

Principal component analysis (PCA) was used to assess item alignment with the intended stages and domains of the SED‐Q. Instead of relying on eigenvalues, factor loadings were assessed to determine if items grouped as proposed. Specifically, the evaluation considered whether items within each stage loaded strongly on the same component and whether stages within the same domain formed cohesive clusters. Loadings above 0.6 were considered strong, 0.4–0.6 moderate and below 0.4 weak. Additionally, whether items for distinct stages loaded onto separate components was examined to evaluate construct distinctiveness (Hair et al. 2009; Kaiser 1960).

Pearson's and Spearman's correlations assessed the relationships and distinctiveness between domains. Correlations < 0.3 indicate distinctiveness, 0.3–0.7 as related but distinct and above 0.7 suggesting potential redundancy. Moderate correlations were deemed satisfactory, as they reflect both the distinctiveness of domains and their relationship with emotional development.

Item analysis evaluated variance (< 0.05 indicating limited variance), discriminative power (> 0.8 indicate highly correlated), correlations (< 0.3 negative‐low correlations) and the internal consistency (Morling 2021; Moses 2017; Nunnally and Bernstein 1994; Verhulst and Neale 2021).

Cronbach's alpha and McDonald's omegas assessed internal consistency. According to Ponterotto and Ruckdeschel (2007), values < 0.60 were classified as unsatisfactory, 0.60–0.69 as fair, 0.70–0.79 as moderate, 0.80–0.89 as good and ≥ 0.90 as excellent.

Convergent validity was assessed by comparing the SED‐Q to the SED‐S using Pearson's and Spearman's correlations, with values > 0.70 indicating strong, 0.50–0.70 moderate and < 0.50 low validity. SED‐Q scores from Stages 6 and 7 were recoded into Stage 5 for alignment. Discriminant validity was tested against the QET, RFQ, AQ10 and subscales, with values < 0.30 indicating strong discriminant validity.

Test–retest reliability was assessed using ICCs from two testing sessions (*k* = 2) with a consistency agreement approach in a two‐way mixed‐effects model. Reliability was classified as poor (< 0.50), moderate (0.50–0.75), good (0.75–0.90) or excellent (> 0.90) (Koo and Li 2016; Morling 2021).

An ANOVA with post hoc tests assessed group differences in emotional development across levels of intellectual disability, autism diagnosis, visual impairment and interviewer experience. Differences between domains were separately analysed for each domain across levels of intellectual disability. Participants unaware of their severity of intellectual disability were excluded.

## 4 Results

### 4.1 PCA

The PCA indicates that many questions across stages had strong factor loadings (> 0.6; 51.79%), indicating alignment with the intended construct and proper functioning. Items with moderate loadings may need refinement to enhance clarity or relevance (0.4–0.6; 30.00%), while those with low loadings (< 0.4; 18.21%) likely require substantial revision.

Examining the variance explained, the first principal component (PC1) for each domain explained 10%–17% of the variance, indicating the stages partially capture the domain's theme to some extent but do not form a strongly cohesive unidimensional construct. Maximum PC1 loadings ranged from 0.27 to 0.36, showing no single item dominated the domain measurement. The second principal component (PC2) explained 7%–11% of the variance, highlighting some differentiation among stages but also some overlap.

### 4.2 Correlations Between Domains

Table 3 displays the correlations between domains revealing varying levels of strength between domains, indicating low to moderate correlations.

**TABLE 3:** Correlations between domains.

Domain pair	Pearson's correlation (95% CI)	Pearson's interpretation	Spearman's correlation (95% CI)	Spearman's interpretation
Domain 1 vs. Domain 2	0.11 (−0.06, 0.27)	Low	0.11 (−0.08, 0.30)	Low
Domain 1 vs. Domain 3	0.21 (0.05, 0.36)	Low	0.15 (−0.03, 0.30)	Low
Domain 1 vs. Domain 4	0.11 (−0.05, 0.28)	Low	0.13 (−0.03, 0.30)	Low
Domain 1 vs. Domain 5	0.10 (−0.08, 0.28)	Low	0.09 (−0.08, 0.27)	Low
Domain 1 vs. Domain 6	0.03 (−0.19, 0.23)	Low	0.07 (−0.11, 0.24)	Low
Domain 1 vs. Domain 7	−0.06 (−0.25, 0.11)	Low	−0.01 (−0.18, 0.16)	Low
Domain 1 vs. Domain 8	0.02 (−0.16, 0.21)	Low	0.02 (−0.16, 0.20)	Low
Domain 2 vs. Domain 3	0.35 (0.20, 0.51)	Moderate	0.37 (0.21, 0.51)	Moderate
Domain 2 vs. Domain 4	0.37 (0.23, 0.53)	Moderate	0.38 (0.22, 0.52)	Moderate
Domain 2 vs. Domain 5	0.16 (0.00, 0.34)	Low	0.16 (−0.01, 0.34)	Low
Domain 2 vs. Domain 6	0.20 (0.06, 0.35)	Low	0.22 (0.07, 0.37)	Low
Domain 2 vs. Domain 7	0.16 (0.03, 0.30)	Low	0.16 (0.02, 0.31)	Low
Domain 2 vs. Domain 8	0.16 (−0.02, 0.32)	Low	0.16 (−0.01, 0.33)	Low
Domain 3 vs. Domain 4	0.26 (0.08, 0.42)	Low	0.30 (0.12, 0.45)	Moderate
Domain 3 vs. Domain 5	0.33 (0.17, 0.47)	Moderate	0.34 (0.19, 0.48)	Moderate
Domain 3 vs. Domain 6	0.25 (0.08, 0.40)	Low	0.24 (0.07, 0.40)	Low
Domain 3 vs. Domain 7	0.17 (−0.05, 0.38)	Low	0.22 (0.04, 0.39)	Low
Domain 3 vs. Domain 8	0.21 (0.01, 0.38)	Low	0.23 (0.07, 0.39)	Low
Domain 4 vs. Domain 5	0.21 (0.06, 0.37)	Low	0.21 (0.05, 0.37)	Low
Domain 4 vs. Domain 6	0.20 (0.04, 0.37)	Low	0.21 (0.03, 0.38)	Low
Domain 4 vs. Domain 7	0.41 (0.28, 0.55)	Moderate	0.35 (0.17, 0.50)	Moderate
Domain 4 vs. Domain 8	0.40 (0.25, 0.55)	Moderate	0.40 (0.25, 0.54)	Moderate
Domain 5 vs. Domain 6	0.29 (0.13, 0.44)	Low	0.27 (0.10, 0.42)	Low
Domain 5 vs. Domain 7	0.34 (0.19, 0.49)	Moderate	0.35 (0.20, 0.49)	Moderate
Domain 5 vs. Domain 8	0.26 (0.09, 0.41)	Low	0.27 (0.10, 0.43)	Low
Domain 6 vs. Domain 7	0.22 (0.03, 0.37)	Low	0.21 (0.03, 0.39)	Low
Domain 6 vs. Domain 8	0.25 (0.06, 0.42)	Low	0.24 (0.05, 0.40)	Low
Domain 7 vs. Domain 8	0.33 (0.18, 0.48)	Moderate	0.30 (0.14, 0.46)	Moderate

### 4.3 Item Analysis

The item analysis identified several items with limited variance (< 0.05; 3.93%), contributing less to the reliability of the questionnaire. Correlations were assessed for both the total questionnaire and its subscales. No highly correlated items (> 0.8) were detected. Several negative and low correlated (< 0.3) items were identified for the total questionnaire, as well as within domains with stronger correlations within the domains.

Cronbach's alphas for the total questionnaire and domains suggest that some items might benefit from reverse scoring or revision. However, reverse scoring does not improve the total scale's internal consistency and can slightly decrease Cronbach's alpha, likely due to its already high reliability and the dilution effect from the extensive number of items.

### 4.4 Internal Consistency

**FIGURE 1:**
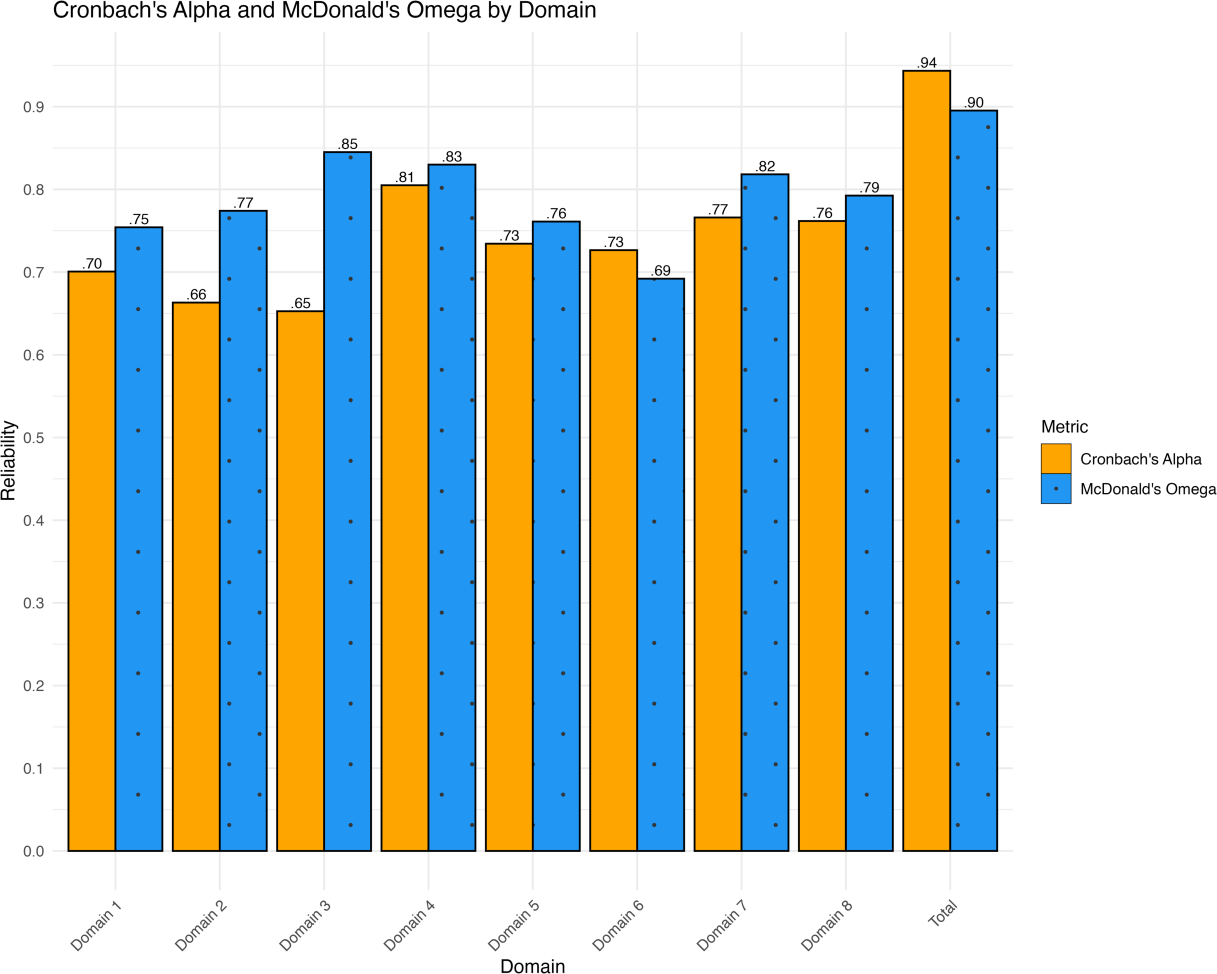
Cronbach's alpha and McDonald's omega by domain.

The SED‐Q demonstrates excellent overall reliability, as indicated in Figure 1 by both Cronbach's alpha and McDonald's omega. The subscales exhibit moderate to good reliability, except for Domains 2 and 3, which show a larger discrepancy between Cronbach's alpha and McDonald's omega. Additionally, Domain 6 displays reliability ranging from fair to moderate.

### 4.5 Test–Retest Reliability

Table 4 displays the 3‐week interval test–retest reliability analysis (*n* = 43) revealing moderate to good reliability.

**TABLE 4:** Three‐week interval test–retest reliability.

Domain	ICC value (95% CI)	ICC interpretation
Domain 1	0.54 (0.16, 0.75)	Moderate reliability
Domain 2	0.55 (0.17, 0.76)	Moderate reliability
Domain 3	0.79 (0.62, 0.89)	Good reliability
Domain 4	0.78 (0.60, 0.88)	Good reliability
Domain 5	0.74 (0.52, 0.86)	Moderate reliability
Domain 6	0.71 (0.46, 0.84)	Moderate reliability
Domain 7	0.64 (0.34, 0.80)	Moderate reliability
Domain 8	0.58 (0.22, 0.77)	Moderate reliability
Total	0.81 (0.65, 0.90)	Good reliability

### 4.6 Convergent Validity

Table 5 represents the convergent validity between the SED‐Q and SED‐S, indicating low convergent validity.

**TABLE 5:** Convergent validity results: correlations between SED‐Q and SED‐S.

Domain	Pearson's correlation (95% CI)	Pearson's interpretation	Spearman's correlation (95% CI)	Spearman's interpretation
Domain 1	0.17 (−0.01, 0.33)	Low convergent validity	0.14 (−0.04, 0.29)	Low convergent validity
Domain 2	0.26 (0.09, 0.43)	Low convergent validity	0.26 (0.09, 0.42)	Low convergent validity
Domain 3	0.26 (0.09, 0.44)	Low convergent validity	0.25 (0.07, 0.41)	Low convergent validity
Domain 4	0.24 (0.05, 0.40)	Low convergent validity	0.23 (0.06, 0.40)	Low convergent validity
Domain 5	0.15 (−0.02, 0.34)	Low convergent validity	0.13 (−0.04, 0.31)	Low convergent validity
Domain 6	0.20 (−0.08, 0.45)	Low convergent validity	0.10 (−0.08, 0.29)	Low convergent validity
Domain 7	0.14 (0.00, 0.30)	Low convergent validity	0.14 (−0.01, 0.31)	Low convergent validity
Domain 8	0.30 (0.12, 0.46)	Low convergent validity	0.31 (0.15, 0.46)	Low convergent validity
Total	0.41 (0.22, 0.58)	Low convergent validity	0.43 (0.26, 0.56)	Low convergent validity

### 4.7 Discriminant Validity

Table 6 represents the discriminant validity between the SED‐Q and the QET, RFQ, AQ‐10 and included subscales, indicating strong discriminant validity.

**TABLE 6:** Discriminant validity results: correlations of SED‐Q with other variables.

Variable	Pearson's correlation (95% CI)	Pearson interpretation	Spearman's correlation (95% CI)	Spearman interpretation
RFQ	0.28 (0.11, 0.43)	Strong discriminant validity	0.28 (0.11, 0.44)	Strong discriminant validity
RFQ self	0.24 (0.06, 0.40)	Strong discriminant validity	0.25 (0.09, 0.41)	Strong discriminant validity
RFQ others	0.23 (0.08, 0.38)	Strong discriminant validity	0.23 (0.07, 0.37)	Strong discriminant validity
AQ10	0.20 (0.02, 0.38)	Strong discriminant validity	0.23 (0.05, 0.39)	Strong discriminant validity
QET	0.09 (−0.08, 0.26)	Strong discriminant validity	0.09 (−0.09, 0.27)	Strong discriminant validity
QET hypervigilance	−0.04 (−0.22, 0.14)	Strong discriminant validity	0.00 (−0.18, 0.17)	Strong discriminant validity
QET curiosity	−0.18 (−0.34, 0.01)	Strong discriminant validity	−0.15 (−0.33, 0.03)	Strong discriminant validity
QET expectation	−0.01 (−0.19, 0.17)	Strong discriminant validity	−0.03 (−0.20, 0.16)	Strong discriminant validity
QET openness	−0.11 (−0.30, 0.07)	Strong discriminant validity	−0.13 (−0.30, 0.06)	Strong discriminant validity

### 4.8 Group Differences

The ANOVA results indicate that people with visual impairments and those with autism score similarly on emotional development compared to their counterparts without these conditions (visual impairment: *F*_1,128_ = 1.66, *p* = 0.200, *η* ^2^ = 0.01; autism: *F*_1,128_ < 0.01, *p* = 0.932, *η* ^2^ < 0.00). Interviewer experience had no significant effect on assessing emotional development (*F*_1,114_ = 1.36, *p* = 0.246, *η* ^2^ = 0.01).

In contrast, people with different levels of intellectual disabilities displayed significant differences in emotional development (*F*_3,127_ = 5.59, *p* = 0.001, *η* ^2^ = 0.12). Post hoc analysis revealed that people with borderline intellectual disability scored significantly higher than those with moderate intellectual disability (*p* < 0.001), and those with mild intellectual disability also differed significantly from those with moderate intellectual disability (*p* = 0.008).

Domain‐specific differences were examined, revealing significant differences in Domains 7 and 8 (*F* = [6.14–6.56], *p* = [0.003–0.002], *η* ^2^ = [0.09–0.10]). Borderline intellectual disability scored higher than moderate in both domains (*p* = 0.010 and *p* = 0.001), and in Domain 8, higher than mild (*p* = 0.032). In Domain 7, mild scored higher than moderate (*p* = 0.002). Borderline differences emerged for Domains 4 and 5 (*F* = [2.89–3.04], *p* = [0.051–0.060], *η* ^2^ = 0.04–0.05), with mild scoring higher than moderate in Domain 5 (*p* = 0.048). No significant pairwise differences emerged for Domain 4, although borderline intellectual disability and moderate intellectual disability approached significance (*p* = 0.052). No differences were found in Domains 1–3 and 6.

### 4.9 Measurement Differences

Table 7 displays the mean differences across SED‐Q and SED‐S domains. SED‐Q scored significantly higher on most domains, with similar scores on Domains 1, 3 and 6.

**TABLE 7:** Mean differences SED‐Q and SED‐S.

Domain	SED‐Q mean	SED‐S mean	*p* value
Domain 1	3.46	3.48	0.869
Domain 2	4.03	3.07	< 0.001**
Domain 3	4.08	3.95	0.224
Domain 4	3.66	2.63	< 0.001**
Domain 5	4.02	3.18	< 0.001**
Domain 6	4.32	4.31	0.897
Domain 7	4.36	3.85	< 0.001**
Domain 8	4.22	3.36	< 0.001**
Total	3.84	3.34	< 0.001**

* *p* < 0.05.

** *p* < 0.001.

## 5 Discussion

To better align support with the emotional and self‐reported needs of persons with an intellectual disability, an adapted version of the SED‐S, the SED‐Q, was developed. This study aimed to explore the psychometric properties of the SED‐Q and identify areas requiring improvement. Specifically, the analysis identified items with low variance, low or negative correlations, low consistency and factor loadings which require further refinement to enhance the internal structure of the questionnaire and increase the explained variance. Most domains were found to be distinct from one another, while a few demonstrated being related but distinct. The SED‐Q demonstrated discriminant validity by effectively distinguishing between mentalising, autism and epistemic trust. However, it did not demonstrate convergent validity with the SED‐S. Test–retest reliability ranged from moderate to good. Group differences in emotional development were evident for intellectual disability levels, though not consistently across all levels of severity or all domains. In contrast, no significant differences were found for autism (unexpected), visual impairments (as expected) or interviewer experience.

The PCA results indicate that while SED‐Q stages capture meaningful aspects of emotional development, item loadings vary in strength. Within each domain, stages relate to the overall construct but do not form a tightly cohesive factor, likely reflecting the SED‐Q's design and the fact that emotional development exists on a continuum (Dosen 2005a). Participants tend to endorse items closely aligned with their emotional development level but not those from distant stages, reducing variation across stages within domains and limiting internal consistency. In contrast, the SED‐S displayed a strong, robust one‐factor structure with consistent high domain loadings (Flachsmeyer et al. 2023). This highlights the need for further refinement of the SED‐Q to strengthen its internal structure.

The item analysis identified potential misalignment between some items and the intended construct. Low‐factor loadings should be revised, aiming for ≥ 0.050, and moderate loadings may still be acceptable, depending on the theoretical relevance (Cambridge 2013; Hair et al. 2009). Negative‐low correlations of items with the construct may not contribute effectively to the construct and should be revised (Nunnally and Bernstein 1994). Items with limited variance should be revised. Items with weak or negative internal consistency, and if removal substantially improves reliability, should be revised (Morling 2021; Verhulst and Neale 2021). Revisions are particularly critical within domains and stages, where a fewer number of items increases the sensitivity to misaligned items, whereas examining the entire questionnaire may obscure item‐level differences in alignment with the construct and reliability.

The SED‐Q demonstrates excellent overall reliability, aligning with previous findings from the SED‐S in an adult sample with intellectual disabilities (Meinecke et al. 2024). Domains demonstrate moderate to good reliability, except for Domains 2, 3 and 6. Discrepancies between Cronbach's alpha and McDonald's omega in Domains 2 and 3 underscore the limitations of Cronbach's alpha in certain contexts. Specifically, McDonald's omega is better equipped to account for unequal item contributions and multidimensionality, providing a more accurate measure of reliability for the SED‐S in this context (Malkewitz et al. 2023). Domain 6 displayed slightly lower reliability, ranging from fair to moderate reliability.

The 3‐week test–retest analysis showed moderate to good reliability across domains, with the total score demonstrating the strongest reliability. However, wide confidence intervals suggest limited accuracy and statistical power.

The low convergent validity between the SED‐Q and SED‐S may reflect differences in subjective perspectives, as proxy reports are often distorted by interpretive biases and may not accurately capture people's own experiences (Elliott et al. 2008; Li et al. 2015). Additionally, reduced capacity for mentalisation may limit self‐awareness (Adrien et al. 2021; Benson 1993). The strong discriminant validity demonstrates that the SED‐S successfully distinguishes emotional development from mentalisation and epistemic trust, which are distinct yet closely interconnected constructs (Fonagy and Allison 2014; Knapen 2024).

To evaluate the functionality of the SED‐Q, statistical methods such as PCA, reliability and validity assessments were used to thoroughly identify areas for improvement. This analysis is further bolstered by the inclusion of a diverse sample, including participants with varying levels of intellectual disabilities, autism and visual impairments, as well as the impact of interviewer experience, highlighting the SED‐Q's applicability across contexts.

Several limitations should be acknowledged. While PCA was conducted, its drawbacks include an overemphasis on variance explained, which may overshadow meaningful data patterns; challenges in practical interpretation; and a lack of consideration for variable context, potentially misrepresenting real‐world relevance (Abdi and Williams 2010; Greenacre et al. 2022; Jolliffe 1990). The uneven score distribution, especially in Stages 1, 6 and 7, made certain analyses unfeasible, while the small sample size limited the detection of significant effects and weakened the robustness of the conclusions. Because of its length, the SED‐Q sometimes caused fatigue and reduced concentration, which led to it being administered in multiple sessions. This may have influenced the results. Additionally, interviewers occasionally needed to simplify or rephrase questions for clarity. To improve clarity, the items were modified but, due to time constraints, were not sufficiently checked with the target group, which needs to be addressed in future developments of the instrument.

Future research should include a larger, more diverse sample to encompass people with various levels of intellectual disabilities, borderline intellectual disabilities and those without intellectual disabilities, as well as revise items with the target group (Nicolaidis et al. 2020). This would enable a thorough investigation of the SED‐Q's psychometric properties and enhance analysis power. Additionally, after improving the items, future research could focus on an in‐depth item analysis similar to that of Hermann et al. (2024) and reliability and validity analysis of Flachsmeyer et al. (2023). In addition to the psychometric evaluations, it would also be valuable to explore the practical application of SED‐Q, including how the interview is experienced, its current use as a descriptive tool in practice where it is already at times employed to gain insight and its potential to foster self‐awareness (DeJonckheere and Vaughn 2019).

In conclusion, the SED‐Q is a promising semi‐structured self‐report interview with a professional to assess emotional development, aiming to better meet the emotional needs of persons with moderate to borderline intellectual functioning, complementing the proxy perspective of the SED‐S. However, revisions are needed, particularly for items with low variance, low or negative correlations, low consistency and low factor loadings, and they should involve the target group to better align with the intended construct and enhance reliability. Following these revisions, future studies should evaluate the revised SED‐Q's psychometric properties, using methods similar to the item analysis of Hermann et al. (2024) and reliability and validity analysis of Flachsmeyer et al. (2023).

## Disclosure

This work does not include any reproduced material from other sources.

## Ethics Statement

The study adhered to the principles of the Declaration of Helsinki (Version 7, October 2013) and United Nations Convention on the Rights of Persons with Disabilities by, where possible, allowing participants to answer the questions about themselves. The Institutional Review Board of the Faculty of Behavioural and Movement Sciences of the Vrije Universiteit Amsterdam granted ethical approval (VCWE‐2022‐140). The study was assessed and found to be exempt from the provisions of the Medical Research Involving Human Subjects Act (WMO; 2022.0466). To minimise potential bias, the questionnaire's creator (J. Vonk) abstained from participating in both the data collection process and the statistical analysis.

## Consent

Participants were asked to read and sign a consent form before the start of the study. For participants with visual disabilities, the form was read out loud. If participants were legally incapacitated regarding giving consent, the agreement was signed by their legal representative. Their supervisors, parents or other involved caregivers were also asked to sign a consent form as they completed a questionnaire about the participant. Additionally, a general information letter was provided to the professional caregiver and legal representative to ensure they could support the participant in making a well‐informed decision. A 2‐week period was provided for participants to consider their participation. Independent researchers, including assistants and students, signed a confidentiality agreement.

## Conflicts of Interest

The authors declare no conflicts of interest.

## Data Availability

The data supporting the findings of this research are available from the corresponding author upon reasonable request.
